# The Promise and Limitations of Metabolic Trait Prediction Across Diverse Bacteria

**DOI:** 10.1111/1462-2920.70478

**Published:** 2026-11-04

**Authors:** Matti Gralka

**Affiliations:** ^1^ Systems Biology Group, Amsterdam Institute for Life and Environment (A‐LIFE) Vrije Universiteit Amsterdam Amsterdam the Netherlands; ^2^ Amsterdam Institute of Molecular and Life Sciences (AIMMS) Vrije Universiteit Amsterdam Amsterdam the Netherlands

## Abstract

Metabolic traits mediate microbial interactions and underpin mechanistic models of community assembly. With the rapid expansion of genomic data and bioinformatic tools, it is widely assumed that metabolic traits such as substrate utilization can be reliably inferred from genomes alone. Here, we systematically evaluate this assumption by comparing genome‐based predictions from four commonly used tools to high‐throughput growth phenotypes of 186 marine bacteria. For simple substrates, prediction accuracy was limited and highly variable across taxa and substrates, with many strains predicted at or near chance levels. For three of four tools, most errors were false positives, indicating that apparent pathway completeness frequently fails to translate into growth under laboratory conditions. In contrast, genomic prediction of polysaccharide utilization based on carbohydrate‐active enzyme content performed substantially better, suggesting that traits that depend on a few highly specific enzymes are easier to predict. While genome‐based approaches can recover broad statistical patterns in metabolic strategies, their limited strain‐level reliability constrains ecological inference and modelling. For the foreseeable future, large‐scale experimental phenotypic characterization will remain an indispensable component of microbial ecology, both to reveal trait distributions and to ground‐truth and improve genomic predictions.

## Graphical abstract

Predicting metabolic capabilities of environmental bacteria holds great promise for microbial ecology, but the reliability of available tools is often unclear. This perspective suggests that phenotype predictions are often inaccurate in surprising ways, which makes experimental phenotyping essential for accurate ecological modelling.

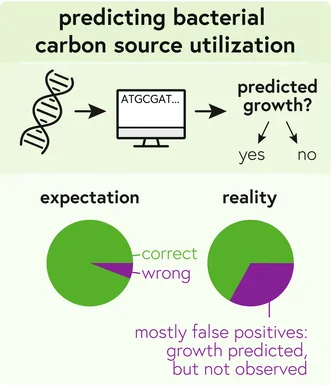

## 1 Introduction

The metabolic traits of heterotrophic microbes, such as growth rates on different substrates, metabolic excretions and biosynthetic capabilities, mediate interactions among species and ultimately shape microbial fitness in community contexts (Kehe et al. 2021; Aranda‐Díaz et al. 2023; Zakem et al. 2025; Reynolds et al. 2023). These traits determine how microbes compete for resources, exchange metabolites and respond to environmental change, and thus form a critical link between microbial physiology and community‐level patterns such as coexistence, resilience and function. Consequently, there has been long‐standing interest in developing trait‐based approaches for heterotrophic microbial communities (Zakem et al. 2025; Rodriguez et al. 2025; Malik et al. 2020; Marschmann et al. 2024; Martiny et al. 2013; Allison 2012), analogous to those that have proven powerful in macroscopic ecology and phytoplankton communities (Litchman and Klausmeier 2008), to interpret community composition data. Beyond interpretation, metabolic traits also play a central role in ecological modelling. In consumer‐resource frameworks, metabolic traits directly parameterize resource uptake, growth and metabolic transformations (van den Berg et al. 2022), allowing species interactions and community dynamics to emerge from the underlying physiology rather than being imposed phenomenologically (Aranda‐Díaz et al. 2023; Niehaus et al. 2019; Ho et al. 2024).

These applications implicitly assume that microbial traits are either known or can be predicted with sufficient accuracy. However, given the immense taxonomic diversity of microbial communities, detailed experimental characterization of all constituent species is infeasible in most systems. The rapid accumulation of genomic and metagenomic data, together with a relatively complete understanding of central metabolic pathways, has fuelled the expectation (or, at least, the hope) that microbial metabolic traits can be inferred directly from genome sequences. Indeed, there have been widespread efforts to predict metabolic traits from genomes alone (Zimmermann et al. 2021; Machado et al. 2018, 2021; Price et al. 2022, 2020; Schäfer et al. 2023; Gralka et al. 2023; Karlsen et al. 2023; Bulka et al. 2026; Linsay et al. 2010). Genomic data are also used to predict metabolite fluxes and interactions in whole communities, either by mapping 16S rRNA amplicon data to genome‐scale metabolic models or directly building models from metagenomes (Machado et al. 2021; Quinn‐Bohmann et al. 2024; Giordano et al. 2024; Li et al. 2025; Baskaran et al. 2023; Guo et al. 2026; Ginatt et al. 2024; Mataigne et al. 2022; Schlechter et al. 2023; Orellana et al. 2025; Zhang et al. 2024). However, despite the conceptual appeal of genome‐based metabolic trait inference, there is an open debate (Price 2023; Machado and Patil 2023) about the extent to which current prediction methods recover microbial phenotypes across diverse, non‐model organisms at a level of accuracy that is meaningful for ecological applications.

Here, we argue that current methods fall well short of this expectation. By systematically comparing genome‐based predictions to experimentally measured phenotypes across diverse bacterial isolates, we show that current approaches are often unreliable at recovering common metabolic traits (here, carbon source utilization). These limitations occur across taxa, substrate classes and tools, suggesting that they reflect general features of microbial metabolism rather than isolated shortcomings of individual tools. This has important consequences for how genome‐derived trait predictions should be interpreted, particularly when applied to environmental datasets. In contrast, traits that depend on a few specific enzymes, such as polysaccharide degradation, and coarse‐grained trait distributions are predicted well.

## 2 What Large‐Scale Trait Characterization Reveals About Microbial Ecology and Biochemical Constraints

Recent high‐throughput phenotyping efforts have mapped microbial metabolic traits systematically across large collections of non‐model organisms and integrated this data with genomic information (Schäfer et al. 2023; Gralka et al. 2023; Forchielli et al. 2022; Li et al. 2023). A central result that emerges from large phenotype databases is the identification of low‐dimensional axes that capture substantial variation in microbial metabolic strategies (Fahimipour and Gross 2020). Rather than occupying an unstructured, high‐dimensional space, bacterial metabolic traits often cluster along ecologically interpretable gradients, reflecting differences in resource use, energy metabolism and investment in biosynthetic capabilities (Rodriguez et al. 2025; Martiny et al. 2013; Sun et al. 2024; Zhu and Dai 2024).

For example, high‐throughput measurements of growth on diverse carbon sources have uncovered consistent patterns across independent strain collections and ecosystems (Kehe et al. 2021; Gralka et al. 2023). One observation is that strains are often distinguished by broad substrate preferences for sugars versus organic acids, suggesting a fundamental metabolic trade‐off in central carbon metabolism (Gralka et al. 2023; Schink et al. 2022). Within these broad substrate classes, substrate utilization is typically uneven and nested: some substrates are widely utilized across taxa, whereas others are rarely catabolized (Flickinger and Gralka 2025). Similarly, large‐scale phenotyping studies of amino acid and vitamin auxotrophies show that auxotrophies are common, metabolite‐specific and often phylogenetically structured (Gregor et al. 2025; Yousif et al. 2026 ). Integrating such patterns with genomic data, environmental metabolomes and biochemical insights can reveal the underlying biochemical, genetic and evolutionary constraints (Martiny et al. 2013; Gralka et al. 2023; Brbić et al. 2016; Barve and Wagner 2013; Walworth et al. 2021), such as trade‐offs between the biosynthetic cost and catabolic pathway length of a substrate (Flickinger and Gralka 2025), its energetic yield, and its environmental availability (Gregor et al. 2025), as well as evolutionary histories of pathway gain and loss (Pollak et al. 2021).

Axes of variation such as the prevalence of auxotrophies or the distribution of metabolic niches provide compact descriptions of microbial strategies that are directly relevant to ecological theory. For example, differences in substrate preferences can inform expectations about competition and coexistence (Kehe et al. 2021; Machado et al. 2021; Giri et al. 2021), while biosynthetic capabilities and auxotrophies shape a species' dependence on community context (Gregor et al. 2025; Oña et al. 2021).

Importantly, these insights arise from the statistical distribution of traits across many taxa, rather than from the accurate knowledge of any single organism's phenotype, highlighting the importance of high‐throughput trait characterizations of diverse strain libraries (Valiya Kalladi et al. 2025). Learning the distributions of metabolic traits is also important for ecological modelling beyond a given ecosystem because empirically derived trait distributions constrain plausible parameter ranges and correlation structures in mechanistic models, such as consumer‐resource frameworks, even when individual species traits are unknown or uncertain (Marsland et al. 2020; Skwara et al. 2023).

## 3 Where Genomic Predictions Are Unreliable

Given both the importance of microbial traits and the vast diversity and limited cultivability of many taxa, an obvious question is whether these traits can be recovered from genomes alone. Indeed, genome‐based prediction of microbial traits such as growth under aerobic or anaerobic conditions, temperature ranges, motility and others has become a successful strategy in microbial ecology (Ramoneda et al. 2024, 2023; Koblitz et al. 2025). However, this confidence is not always justified for metabolic traits such as substrate utilization. To illustrate this point, we evaluated the performance of four commonly used tools (see Section 7) to predict growth capabilities of diverse bacteria.

### 3.1 Predicting Carbon Source Utilization From Genomes

To test how well genome‐based methods predict carbon source use, we compared predictions to a published high‐throughput phenotyping dataset from Gralka et al. (2023), which quantified growth of 186 marine bacterial isolates on 140 individual carbon sources in minimal media supplemented with vitamins. Focusing first on simple carbon substrates for which metabolic pathways are well‐described, we generated predictions for each isolate‐substrate pair using four widely used, automated tools: GapMind (Price et al. 2022), CarveMe (Machado et al. 2018), GapSeq (Zimmermann et al. 2021) and KBase (Linsay et al. 2010). These tools attempt to infer pathway completeness by identifying metabolic enzymes and assessing pathway completeness using a direct rule (GapMind) or by automatically generating a genome‐scale metabolic model (GEM) that can be queried for growth on a given carbon source (CarveMe, GapSeq, KBase). Each tool uses different databases to map against and, for the GEM creation tools, different algorithms to create the metabolic model and fill perceived gaps in metabolic pathways and can therefore lead to different predictions (Hsieh et al. 2024). We used these tools to predict the ability of a strain to use a single substrate as a sole carbon and energy source. Notably, all tools predict pathway completeness, not necessarily growth on a given substrate as the sole carbon and energy source. We did not test the ability to utilize multiple carbon sources in combination since data of this kind are unavailable to our knowledge. Therefore, it is possible that some substrates can be used in conjunction with others, just not as the sole carbon source; such cases will give rise to incorrect predictions here.

In terms of accuracy, the fraction of correct predictions, GapMind correctly predicted growth phenotypes in 69% of cases, while the GEM‐based approaches achieved accuracies of 61% (GapSeq), 59% (CarveMe) and 70% (KBase), respectively (Figure 1). However, this number alone is misleading, since the growth matrix is sparse, giving undue weight to (correct) negative predictions, especially for KBase. As a more informative null model, we shuffled the measured phenotypes across carbon sources within each strain, thus keeping the number of substrates on which each strain grows. A tool therefore beats the null only if it predicts which substrates a strain uses, not only how many (Section 7). All models performed significantly better than this null model expectations for overall correct predictions (between 44% and 54%) as well as other measures of model accuracy (balanced accuracy, MCC, Figure 1a). Nevertheless, despite performing better than the null model, the overall predictive performance was still limited relative to the naïve intuition that the simplicity of the experimental conditions and the profound understanding of central metabolic pathways should make carbon source utilization straightforward to predict.

**FIGURE 1:**
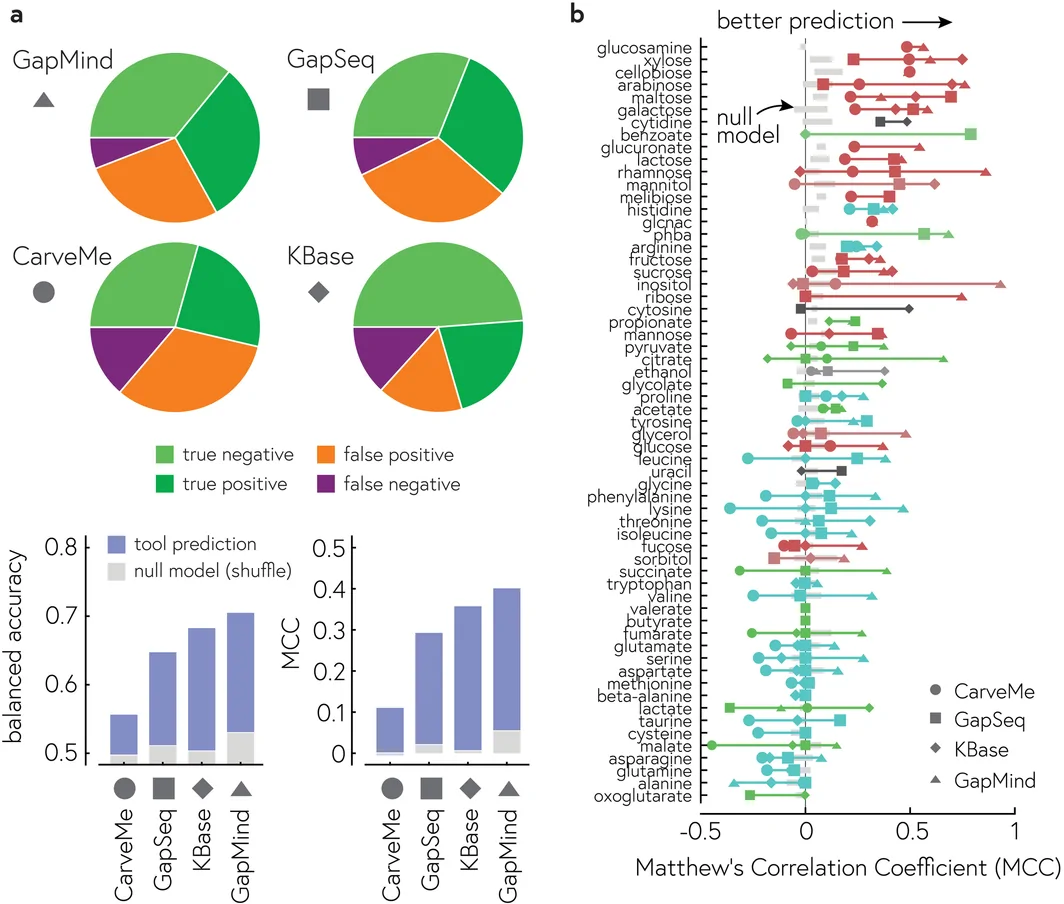
Prediction of utilization of simple carbon sources using four genome‐based tools. (a) All tools show a relatively low average fraction of correct predictions and a large fraction of false positives. Prediction performance, quantified by balanced accuracy and Matthews correlation coefficient (MCC, explained below and defined in methods) varied between tools. (b) Prediction performance (MCC) varied widely by substrate. Grey bars show the range of null model expectation for each carbon source across the four tools. Balanced accuracy runs from 0 to 1, with random chance giving 0.5; MCC runs from −1 to 1, where 1 corresponds to perfect predictions, 0 to no predictability, and negative numbers to worse than chance predictions.

### 3.2 Prediction Performance Varies Strongly Across Clades and Substrates

Inspection of predictions at the level of individual carbon sources revealed pronounced heterogeneity in predictive performance between substrates and prediction tools. Some substrates were predicted well by one or more methods (e.g., glucosamine, cellobiose), while others consistently yielded poor results across all tools (many amino and organic acids, Figure 1b). A striking example is methionine, which supported growth of only a single strain out of 186 isolates, yet was predicted to support growth in a large fraction of strains (Figure S1). Such discrepancies indicate that apparent pathway completeness frequently does not correspond to functional growth capacity. In several cases, different approaches yielded widely varying predictions—for example, accurate predictions for inositol by one tool coincided with poor performance by others—highlighting methodological rather than biological differences in at least a subset of cases.

Similarly, a deeper look into model performance across phylogenetic groups showed that, while some orders showed systematically better‐than‐average performance, every major taxonomic group included strains for which predictions performed at or below the null model expectation (Figure S2). In addition, while some tools had consistent performance across clades, others showed reduced performance in individual orders (Figure S2).

Together, these findings show that these tools should be used carefully when making predictions about individual strains/MAGs and substrates: genome‐based predictions may be informative on the population level (averaging across many strains), but they can sometimes operate at or below chance level for individual strains.

### 3.3 False Positives Dominate the Errors

Across substrates and prediction methods, we observed a mixture of false negatives—cases where growth occurred despite no predicted pathway—and false positives, where growth was predicted but not observed experimentally (Figure 1a). False negatives may plausibly arise from missing annotations or incomplete reference databases, issues that could be mitigated by improved homology detection or expanded biochemical knowledge. False positives, by contrast, are more difficult to reconcile with purely technical explanations and suggest that additional biological factors intervene between genotype and phenotype. Notably, most prediction errors were false positives (except for KBase), indicating that strains often harbour apparently complete metabolic pathways for substrates they cannot catabolize under laboratory conditions in minimal media. We speculate on several non‐mutually exclusive mechanisms in the Discussion.

## 4 Improved Predictability of Polysaccharide Utilization

While genomic prediction of growth on simple substrates performed relatively poorly, we hypothesized that traits mediated by highly specific enzymes might be more amenable to genomic inference. Polysaccharide degradation often depends on carbohydrate‐active enzymes (CAZymes) with narrowly defined substrate specificities, and in many cases a single enzyme family is sufficient to initiate degradation.

To test this hypothesis, we re‐analysed growth data from Gralka et al. (2023), which also quantified bacterial growth on different polysaccharides and annotated corresponding genomes for CAZyme content (Section 7). We predicted growth capability based on the presence of CAZyme families with known substrate ranges (Section 7).

Genomic inference of polysaccharide utilization performed substantially better than for simple substrates, with an average balanced accuracy of 80% (Figure 2), high MCC values and a more balanced proportion of false positives and negatives. For all but one substrate, predictions significantly exceeded null model predictions (MCC), and in several cases, the presence of a single CAZyme family was sufficient to accurately predict growth. These results demonstrate genomic predictability depends strongly on how directly a phenotype maps onto specific genetic components.

**FIGURE 2:**
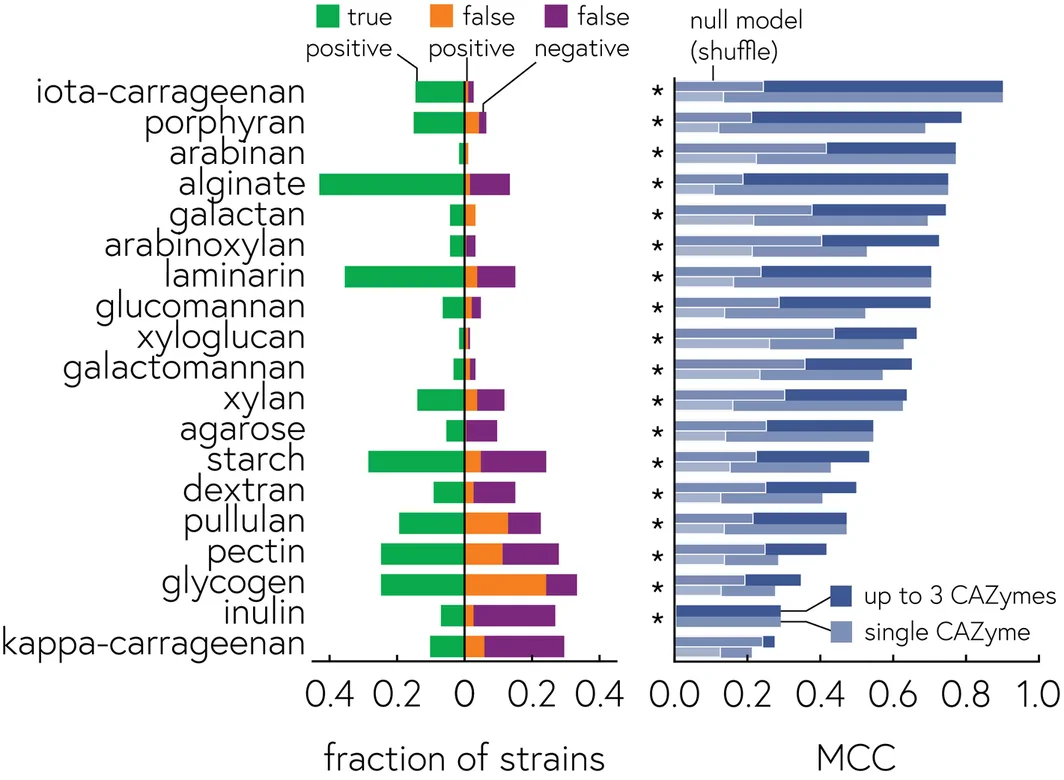
Assessing predictive power for growth on polysaccharides via MCC, as in Figure 1. Predictions based on either a single or up to three CAZymes generally outcompete the null model (randomized data, lighter bars; * indicates significantly better predictions than the null model).

## 5 Discussion

By comparing genome‐based predictions to large‐scale experimental phenotyping data, we have shown that current approaches frequently fail to predict common microbial metabolic traits across diverse, non‐model bacteria even under highly controlled laboratory conditions. Prediction accuracy for simple carbon sources was often only marginally better than chance and varied strongly across substrates and taxa (Figure 1). Our findings thus challenge the common assumption that genome‐based metabolic trait inference is already sufficiently accurate for broad ecological use, especially concerning traits relating to central carbon metabolism. Since these traits also underlie the prediction of species interactions mediated by competition and crossfeeding, such predictions should be used with caution if no experimental validation is available—notably, predictions are more reliable when curated models are used (Schäfer et al. 2023; Quinn‐Bohmann et al. 2024).

### 5.1 Why a Complete Pathway Does Not Guarantee Growth

A consistent feature across prediction methods was the predominance of false‐positive predictions: cases where genomes appeared to encode complete pathways for substrates that did not support growth experimentally (for GapMind, CarveMe and GapSeq; for KBase, false positives and false negatives were similarly common, Figure 1a). While false negatives may arise from missing annotations or incomplete databases, false positives may point to deeper biological constraints on inference. One source of false positives is gap‐filling during GEM creation, which can add reactions that make a pathway appear complete and has previously been shown to lead to such errors in CarveMe predictions (Li et al. 2023; Reynolds et al. 2026). Here, however, this is arguably not the main reason, since false positives were also common for GapMind, which relies on simple pathway mapping without gap‐filling; and while two GEM tools produced more false positives (CarveMe and GapSeq) than GapMind after gap‐filling, KBase did not. For GapSeq, the fraction of false positives was similar without gap‐filling (31%) and with gap‐filling (35%). In addition, for some tools, gap‐filling was strictly required for many strains to produce working models at all, so it is often not possible to omit gap‐filling.

Beyond such technical causes, both the experimental design and bacterial physiology can produce apparent false positives. A key limitation of this analysis is that we did not test combinations of carbon sources or different media combinations. It is possible that some strains can utilize some carbon sources only in combination with others. Indeed, many strains appear to increase their overall carbon uptake in the presence of multiple carbon sources, leading to increased interspecies competition in more nutritionally complex environments (Ono et al. 2025; Shalev et al. 2026). Furthermore, although all tested strains grew in minimal media with at least one tested carbon source (excluding potential amino acid auxotrophies), it is nevertheless possible that some strains require certain cofactors to utilize some carbon sources (Baichman‐Kass et al. 2026). Such physiological effects are currently poorly understood and can bias genome‐based inferences of metabolic traits.

Substrate uptake is a further candidate. Transporter annotation is less reliable than enzyme annotation, and a transporter assigned by homology may have a different substrate specificity. A catabolic pathway can therefore appear complete although the substrate cannot enter the cell. An alternative reason for false positives could be gene regulation preventing pathway expression under experimental conditions (Zampieri et al. 2023), even when the underlying enzymatic machinery is present. Genes within catabolic pathways may also be rendered non‐functional by pseudogenization or regulatory mutations, effectively disabling the pathway without removing its genomic footprint, making this process difficult to detect. Partial or reversible pathway inactivation by regulatory mutations could even represent an evolutionary strategy that enables rapid metabolic innovation by reactivating latent capabilities with just a few mutations (Barve and Wagner 2013). These hypotheses are testable, for example by measuring expression of predicted pathways on non‐growth substrates, or by testing whether short laboratory evolution experiments recover growth on such substrates.

### 5.2 Highly Specific Enzymes Enable Accurate Predictions

Genome‐based predictions perform much better for some phenotypic traits than others; the deciding factor may be the specificity of the enzymes involved. That polysaccharide utilization is more accurately predicted than simple substrates is consistent with the fact that a single enzyme is sufficient to initiate the degradation of many polysaccharides (although some polysaccharides require many enzymes to act in parallel, Sichert et al. 2020). Simple substrates, in contrast, often enter central carbon metabolism through short pathways whose enzymes are shared with other pathways or act on several substrates. Their presence in a genome may therefore say little about whether a given substrate is catabolized. Another example of high predictability is nitrate and nitrite reduction (Gowda et al. 2022), which similarly depends on a few specific enzymes. In that case, not only phenotypic traits, but also interactions between strains could be accurately predicted; even environmental dependencies are directly reflected in genome content (Crocker et al. 2024).

### 5.3 Genomes Predict Trait Distributions Better Than Individual Traits

Predictions were generally unreliable at the level of individual strains, including within taxonomic groups where average performance was overall good (Figure S2). The low reliability at the strain level limits the usefulness of genomic predictions for many ecological applications, including mechanistic modelling and inference of species interactions, which depend on individual phenotypes rather than population averages. The same limitation applies to community‐scale models that map amplicon or metagenomic data onto automatically reconstructed GEMs unless the underlying models are curated.

However, even if individual predictions are error‐prone, genome‐based predictions can be useful when the focus is not on individual strain‐phenotype predictions, but on large‐scale statistical patterns in trait distributions across taxa, potentially at a coarse‐grained level. For example, maximal growth rates and optimal temperatures are relatively well‐predicted across thousands of strains (Weissman et al. 2021; Barnum et al. 2024). Similarly, sugar‐acid preferences (SAP) can be predicted directly from genome content, even when individual phenotypes are not (Gralka et al. 2023). Notably, direct prediction of SAP from a defined set of catabolic genes yields a better prediction ($R^{2}=0.72$) than the more mechanistic tools assessed here (from $R^{2}$ from 0.04 to 0.61, Figure S3). This is in line with previous findings showing that statistical predictions of metabolic traits from genomes can be more accurate than predictions based on mechanistic knowledge of the pathways involved (Li et al. 2023). Such statistical predictions of trait distributions, even when individual traits exhibit uncertainty, are valuable because they can suggest biochemical and ecological constraints and provide reasonable starting points for mechanistic community models, thus providing a foundation for linking microbial physiology to emergent community dynamics.

Finally, what matters for accurate mechanistic predictions of community assembly, and especially, community responses to environmental change are not only qualitative traits like the ability to utilize some carbon source, but also quantitative microbial traits (e.g., substrate‐specific growth rates, lag times) and trait variability with the environment (e.g., changes in temperature or pH). These traits can often evolve rapidly and vary tremendously even between closely related strains of the same species. Therefore, unlike maximal growth rates, these traits may be exceedingly difficult to predict from genomes alone, especially for environmental bacteria without closely related model organisms. Nevertheless, recent work has shown that community‐level function (rather than strain‐level phenotypes) can be predicted with surprising accuracy. These approaches predict community function directly from strain presence‐absence data (Skwara et al. 2023; Diaz‐Colunga et al. 2024) or higher‐dimensional representations of strain phenotypes inferred from genomes (Qian et al. 2026), offering an alternative route towards predictability in microbial ecology.

## 6 Outlook

While different tools can differ in their predictions, the overall limitations of metabolic trait predictions are not specific to individual tools, suggesting that further refinements of existing approaches or databases are unlikely to fully resolve the genotype–phenotype gap in the near term. Thus, improving genomic trait inference may require new approaches that move beyond direct pathway annotation. Large language models (protein language models, genome foundation models, etc.) and other machine‐learning methods appear well‐suited for this task, moving away from strictly mechanistic to annotation‐free, statistical predictions of phenotypes (Nguyen et al. 2024; Northen et al. 2026; Miguel and Zingaretti 2019; James et al. 2025). For example, newly developed computational methods exploiting evolutionary correlations in genome content (Pollak et al. 2021; Wiatrak et al. 2025) perform well on a limited set of metabolic traits, but carbon source utilization predictions are still sparse—likely a consequence of still relatively limited systematic datasets that are required for statistical predictions (Li et al. 2023). Because most prediction errors are false positives, such datasets must include negative results, that is, substrates that were tested but did not support growth, which are rarely reported. Standardized, openly available phenotype datasets that span diverse taxa and conditions would also allow new methods to be benchmarked against the same ground truth. Predictions may also improve when regulatory and environmental context is added, for example by constraining metabolic models with (meta)transcriptomic data (Zampieri et al. 2023) or by learning how environmental conditions shape gene content and interactions (Crocker et al. 2024). For the foreseeable future then, experimental characterization of diverse taxa will remain a crucial component of understanding microbial communities through the lens of metabolic traits by revealing trait distributions and constraints and supplying ground‐truth data for benchmarking and improving genomic inference.

## 7 Methods

### 7.1 Phenotyping Experiment

All experimental data was previously collected and published in Gralka et al. (2023). Briefly, 186 bacteria strains isolated from marine particles were grown in minimal media supplemented with one of 140 individual carbon sources, including various sugars, organic and amino acids and polysaccharides. We analysed the growth data for those 19 polysaccharides for which there was observable growth in at least 5 strains and scored growth on a given substrate as a maximum OD above a threshold value of 0.08.

### 7.2 Statistics

Null model expectation: to calculate a null expectation for model performance (i.e., ‘chance level’ of correct model predictions), we randomized the carbon source labels per strain and computed accuracy measures; shown are the averages across 50 independent randomizations. A tool was considered significantly better than the null model if its score exceeded the scores of all 50 randomizations (*p* < 0.02).

Prediction accuracy was computed using two scores based on (T/F)(P/N) = true/false positive/negative:

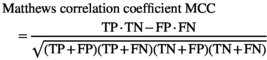

$$\text{Balanced accuracy}=\frac{1}{2}\left(\frac{\mathrm{TP}}{\mathrm{TP}+\mathrm{FN}}+\frac{\mathrm{TN}}{\mathrm{TN}+\mathrm{FP}}\right)$$

### 7.3 Predicting Metabolic Traits From Genomes

Most predictions of metabolic growth phenotypes rely on one of two approaches: direct pathway annotation or the construction of GEMs. Direct pathway annotation aims to identify the presence or absence of genes required for a specific metabolic capability. GEMs are constructed by mapping annotated genes onto large reaction networks and using optimization‐based methods to predict growth under specified conditions. A variety of automated pipelines exist, including CarveMe, GapSeq and KBase, which differ in their underlying assumptions, gap‐filling strategies and treatment of incomplete annotations. In principle, GEMs offer a unified framework for predicting both individual phenotypes and metabolic interactions, but in practice they often require extensive manual curation to achieve high predictive accuracy.

We used four different methods for predicting growth on simple substrates. For genome‐scale metabolic models, we used a minimal media formulation representative of the one used in the experiments during model creation and gap‐filling. Specifically, we provided NH_3_ as a nitrogen source and glucose and pyruvate as potential carbon sources since experimentally, all strains grew on at least one of these carbon sources.

- GapMind (Price et al. 2022): Genomes were run through GapMind to predict pathway completeness for 50 carbon sources. The highest degree of predictability was achieved when considering only pathways with “high” confidence as complete.
- CarveMe (Machado et al. 2018): Genome‐scale metabolic models were generated with CarveMe 1.6.1 with gap‐filling on the minimal media. Although building consensus models across multiple GEM generation runs can produce more reliable models on a molecular level (Reynolds et al. 2026), only a single run of model creation was performed. This choice was made because the expected gain in accuracy is limited since phenotypes are expected to be relatively robust across model ensembles (Machado et al. 2018).
- GapSeq (Zimmermann et al. 2021): Genome‐scale metabolic models were generated with gapseq 1.2 on standard settings. Then, gap‐filling was performed on the resulting draft model with the minimal media.
- KBase (Linsay et al. 2010): Genome‐scale metabolic models were created using KBase *fba_tools* v.2.2.1 with gap‐filling with the minimal media.

GEMs were analysed in python (cobrapy, Ebrahim et al. 2013) or R (cobrar, Waschina n.d.) for growth on a variety of substrates by providing the model with the minimal media amended with a given carbon source (GapSeq: 57 substrates, CarveMe: 51 substrates, KBase: 57 substrates). If the growth objective was non‐zero, we counted this as a positive growth prediction. GEMs that yielded growth on fewer than three substrates were excluded from the analysis.

### 7.4 Predicting Growth on Polysaccharides

The genomes of all strains used in the phenotyping were annotated with dbCANv3 to identify CAZymes (Zheng et al. 2023). The potential activity of each CAZyme was extracted from the substrate mapping file available from dbCAN, selecting all GHs that mention a given polysaccharide by name. From the resulting list of CAZymes per polysaccharide, all possible subsets of length up to three were assembled and checked against the growth phenotypes—if a given strain contained at least one of the GHs in the subset, the growth prediction was positive; otherwise, negative. This simplistic approach has the potential to overestimate growth predictions where multiple enzymes are required to degrade a polysaccharide. However, we observed cases of high false‐positive rates also for simple polysaccharides like starch that can be degraded by a single enzyme.

To quantify the quality of the prediction, we computed the MCC for the simple substrates. As a null model, we randomized the phenotypes and computed MCC again for the gene set that yielded the highest score. Significance was computed as the proportion of MCCs—estimated by deriving an empirical CDF from the randomized MCCs—in the null model higher than the observed, corrected for multiple testing using the FDR correction. We performed this procedure both for the single most predictive genes (subsets of length 1) and for subsets of length up to 3.

## Author Contributions

**Matti Gralka:** conceptualization, investigation, writing – original draft, writing – review and editing, visualization, methodology, formal analysis.

## Conflicts of Interest

The author declares no conflicts of interest.

## Supporting information


**Figure S1:** Fraction of correct and false predictions across carbon sources for the four prediction tools used.
**Figure S2:** Balanced accuracy across substrates, stratified across the five main taxonomic orders in the dataset. Chance levels (averages over null models via shuffled carbon sources) per order are indicated as grey dashed lines. Prediction performance varied by tool between orders (e.g., GapSeq and GapMind perform well on Rhodobacterales, where CarveMe relatively underperforms). For all tools, all orders have representatives that are predicted at or below chance level. Some tools perform well across all clades, while others have more varied performance.
**Figure S3:** Predictions for sugar‐acid preferences across 186 bacterial isolates evaluation across tools, including the pathway‐agnostic model from Gralka et al. (2023). For the pathway‐based tools from the main text, SAP was estimated from the predicted growth on *S* sugars and *A* acids as (*S* − *A*)/(*S* + *A*).

## Data Availability

The data that support the findings of this study are openly available in Mendeley data at https://data.mendeley.com/datasets/xfh8t8568g/5, doi:10.17632/xfh8t8568g.5.
