# Supplementary material for: The Promise and Limitations of Metabolic Trait Prediction Across Diverse Bacteria
Source: Environ Microbiol. 2026 Nov 4;28(11):e70478. doi: 10.1111/1462-2920.70478 (PMC13733489; doi:10.1111/1462-2920.70478)
Supplement: Supplementary file 1 [file EMI-28-e70478-s001.pdf]

Supplementary figures for: The promise and limitations of metabolic trait prediction across diverse bacteria

Matti Gralka[1,2]

[1] Amsterdam Institute for Life and Environment (A-LIFE), Vrije Universiteit Amsterdam, 1081 BT Amsterdam, The Netherlands, [2] Amsterdam Institute of Molecular and Life Sciences (AIMMS), Vrije Universiteit Amsterdam, 1081 BT Amsterdam, The Netherlands

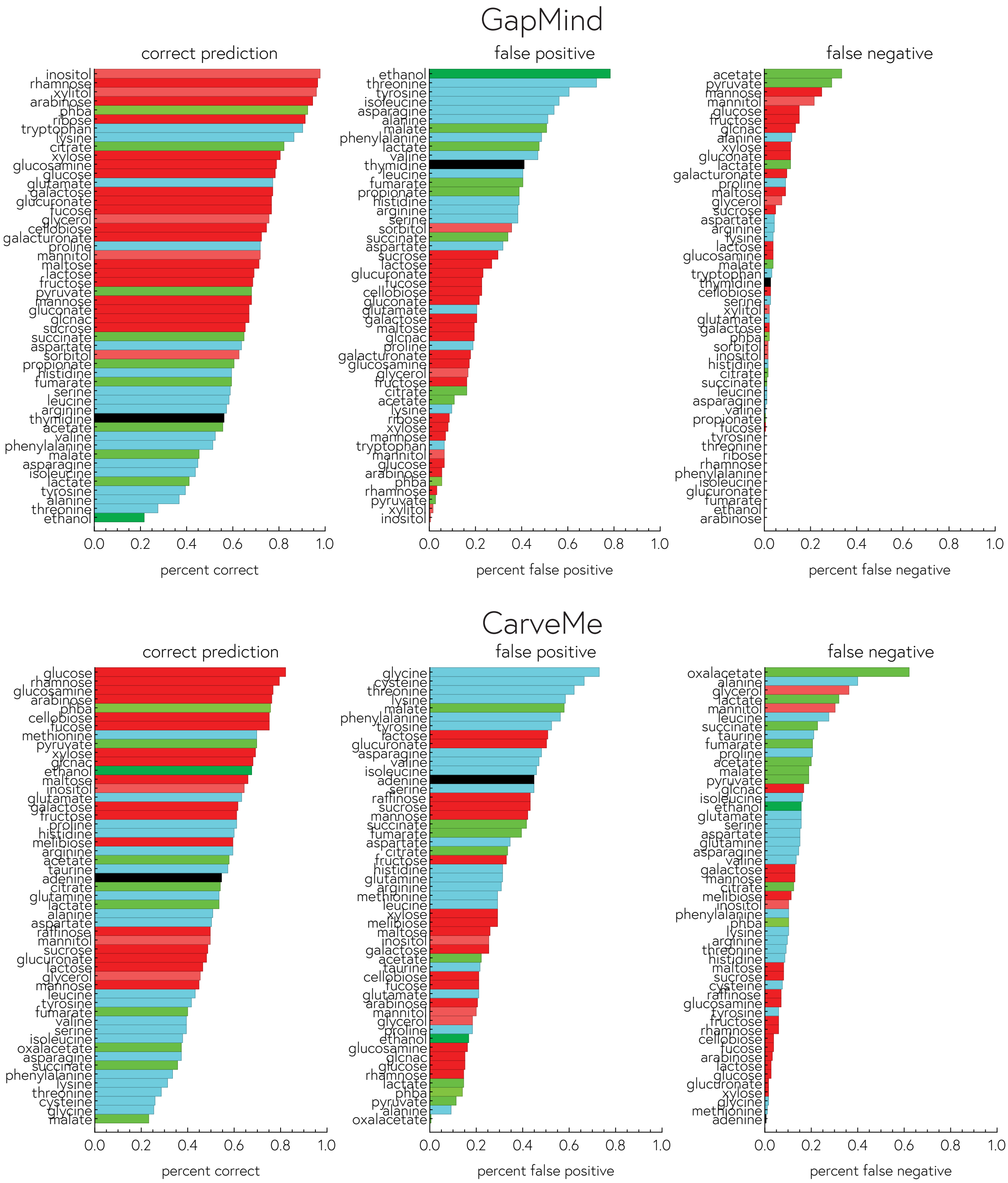
GapMind
correct prediction
inositol
rhamnose
xylitol
arabinose
phba
ribose
tryptophan
lysine
citrate
xylose
glucosamine
glucose
glutamate
galactose
glucuronate
fucose
glycerol
cellobiose
galacturonate
proline
mannitol
maltose
lactose
fructose
pyruvate
mannose
gluconate
glcnac
sucrose
succinate
aspartate
sorbitol
propionate
histidine
fumarate
serine
leucine
arginine
thymidine
acetate
valine
phenylalanine
malate
asparagine
isoleucine
lactate
tyrosine
alanine
threonine
ethanol
0.0 0.2 0.4 0.6 0.8 1.0
percent correct
false positive
ethanol
threonine
tyrosine
isoleucine
asparagine
alanine
malate
phenylalanine
lactate
valine
thymidine
leucine
fumarate
propionate
histidine
arginine
serine
sorbitol
succinate
aspartate
sucrose
lactose
glucuronate
fucose
cellobiose
gluconate
glutamate
galactose
maltose
glcnac
proline
galacturonate
glucosamine
glycerol
fructose
citrate
acetate
lysine
ribose
xylose
mannose
tryptophan
mannitol
glucose
arabinose
phba
rhamnose
pyruvate
xylitol
inositol
0.0 0.2 0.4 0.6 0.8 1.0
percent false positive
false negative
acetate
pyruvate
mannose
mannitol
glucose
fructose
glcnac
alanine
xylose
gluconate
lactate
galacturonate
proline
maltose
glycerol
sucrose
aspartate
arginine
lysine
lactose
glucosamine
malate
tryptophan
thymidine
cellobiose
serine
xylitol
glutamate
galactose
phba
sorbitol
inositol
histidine
citrate
succinate
leucine
asparagine
valine
propionate
fucose
tyrosine
threonine
ribose
rhamnose
phenylalanine
isoleucine
glucuronate
fumarate
ethanol
arabinose
0.0 0.2 0.4 0.6 0.8 1.0
percent false negative
CarveMe
correct prediction
glucose
rhamnose
glucosamine
arabinose
phba
cellobiose
fucose
methionine
pyruvate
xylose
glcnac
ethanol
maltose
inositol
glutamate
galactose
fructose
proline
histidine
melibiose
arginine
acetate
taurine
adenine
citrate
glutamine
lactate
alanine
aspartate
raffinose
mannitol
sucrose
glucuronate
lactose
glycerol
mannose
leucine
tyrosine
fumarate
valine
serine
isoleucine
oxalacetate
asparagine
succinate
phenylalanine
lysine
threonine
cysteine
glycine
malate
0.0 0.2 0.4 0.6 0.8 1.0
percent correct
false positive
glycine
cysteine
threonine
lysine
malate
phenylalanine
tyrosine
lactose
glucuronate
asparagine
valine
isoleucine
adenine
serine
raffinose
sucrose
mannose
succinate
fumarate
aspartate
citrate
fructose
histidine
glutamine
arginine
methionine
leucine
xylose
melibiose
maltose
inositol
galactose
acetate
taurine
cellobiose
fucose
glutamate
arabinose
mannitol
glycerol
proline
ethanol
glucosamine
glcnac
glucose
rhamnose
lactate
phba
pyruvate
alanine
oxalacetate
0.0 0.2 0.4 0.6 0.8 1.0
percent false positive
false negative
oxalacetate
alanine
glycerol
lactate
mannitol
leucine
succinate
taurine
fumarate
proline
acetate
malate
pyruvate
glcnac
isoleucine
ethanol
glutamate
serine
aspartate
glutamine
asparagine
valine
galactose
mannose
citrate
melibiose
inositol
phenylalanine
phba
lysine
arginine
threonine
histidine
maltose
sucrose
cysteine
raffinose
glucosamine
tyrosine
fructose
rhamnose
cellobiose
fucose
arabinose
lactose
glucose
glucuronate
xylose
glycine
methionine
adenine
0.0 0.2 0.4 0.6 0.8 1.0
percent false negative

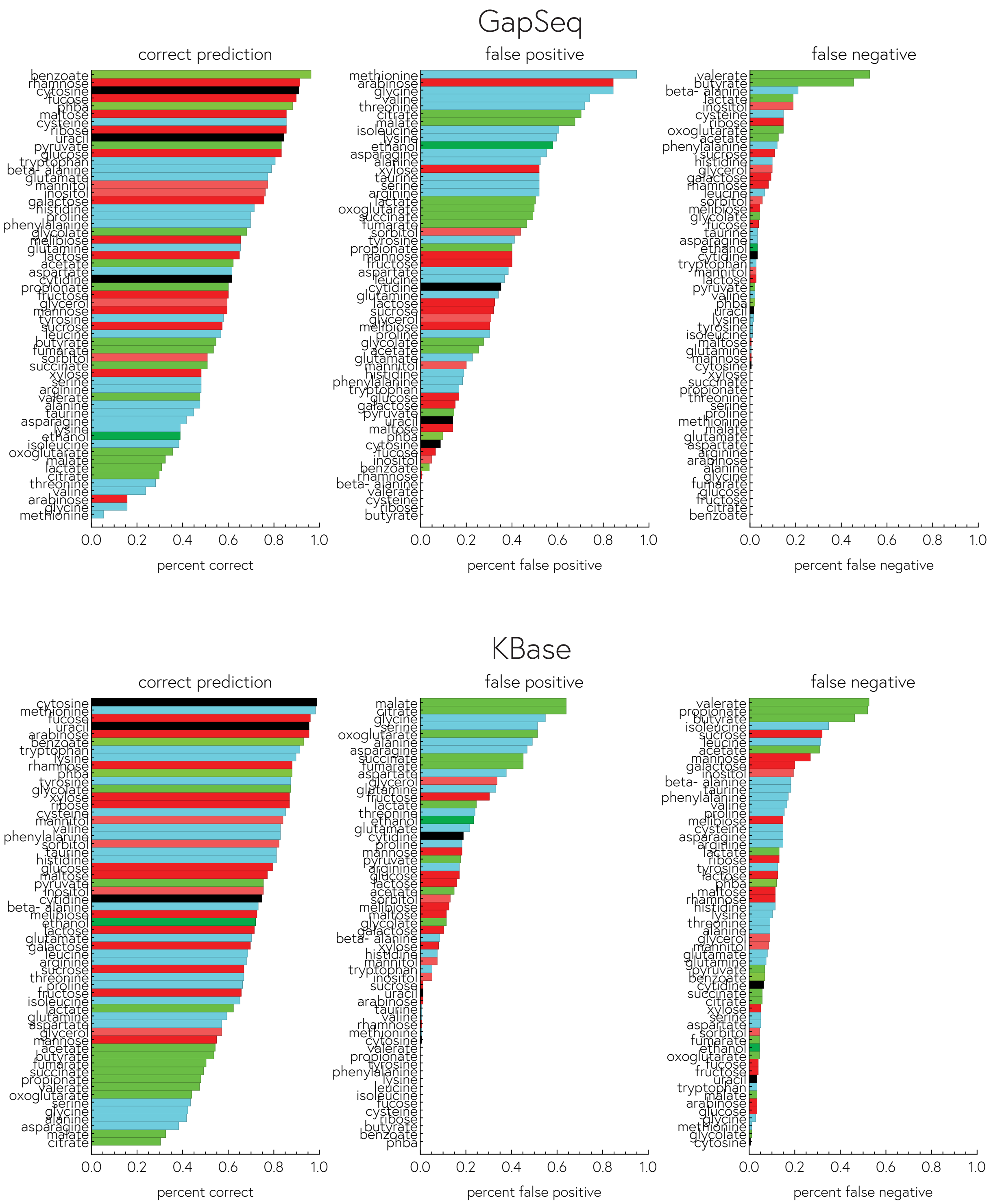

Supplementary Figure 1: Fraction of correct and false predictions across carbon sources for the four prediction tools used.

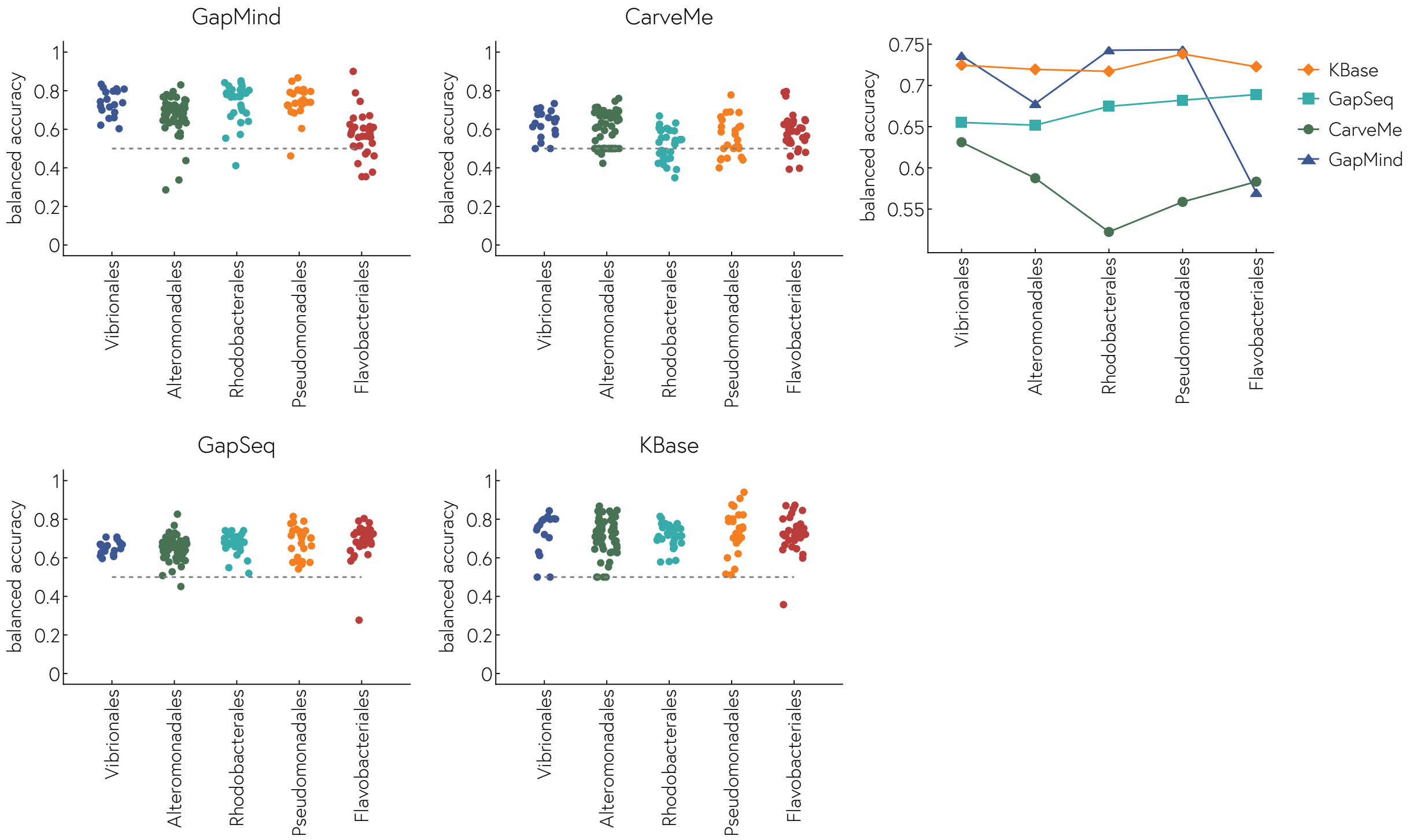

Supplementary Figure 2: Balanced accuracy across substrates, stratified across the five main taxonomic orders in the dataset. Chance levels (averages over null models via shuffled carbon sources) per order are indicated as gray dashed lines. Prediction performance varied by tool between orders (e.g., GapSeq and GapMind perform well on Rhodobacterales, where CarveMe relatively underperforms). For all tools, all orders have representatives that are predicted at or below chance level. Some tools perform well across all clades, while others have more varied performance.

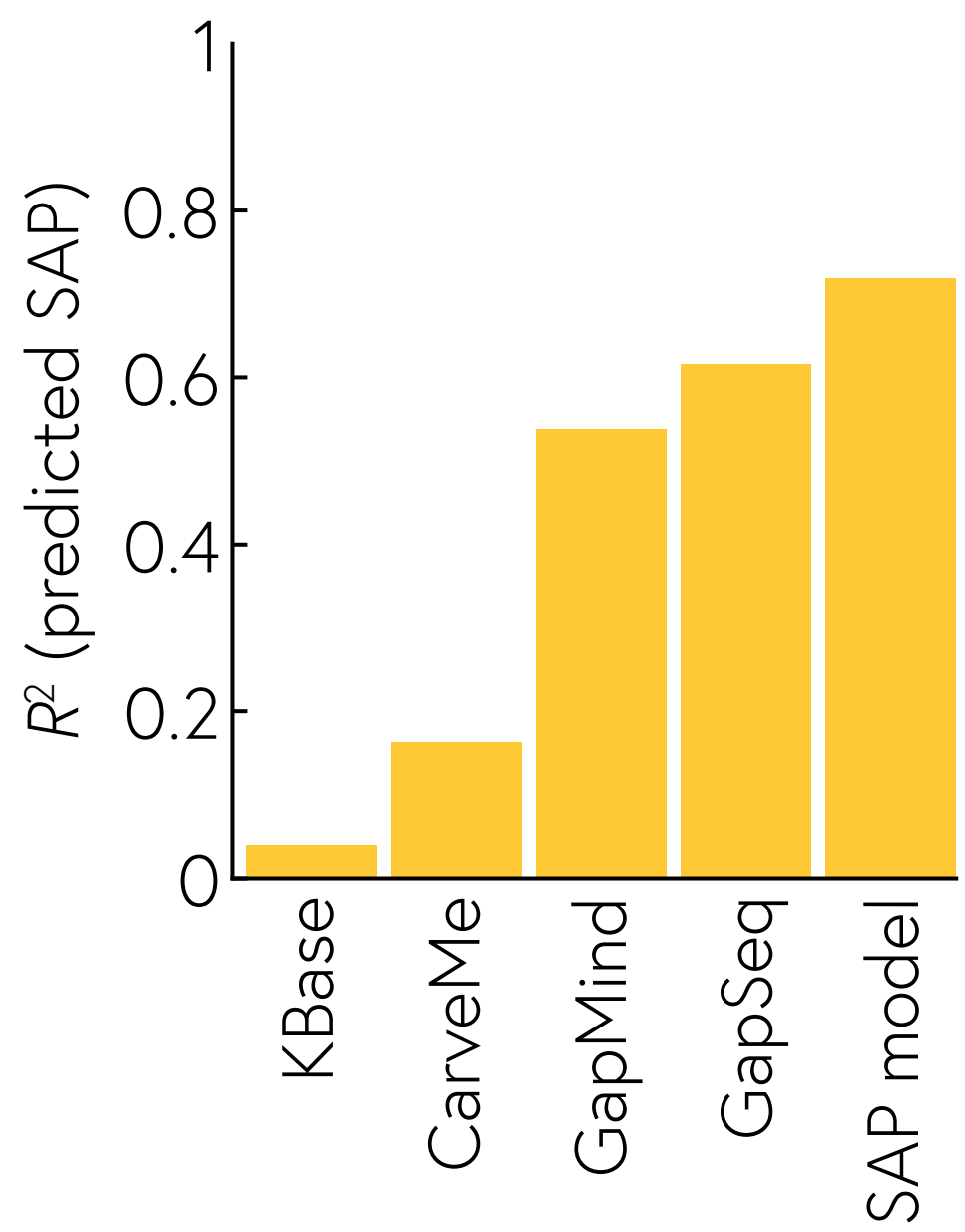

Supplementary Figure 3: Predictions for sugar-acid preferences across 186 bacterial isolates evaluation across tools, including the pathway-agnostic model from Ref. 20. For the pathway-based tools from the main text, SAP was estimated from the predicted growth on S sugars and A acids as $(S - A)/(S + A)$.
